# Progress of Surgery

**Published:** 1874-05

**Authors:** Jno. E. Owens

**Affiliations:** Lecturer on Surgery, Rush Medical College, Chicago


ARTICLE I.—*Progress of Surgery.* By JNO. E. OWENS, M.D., Lecturer on Surgery, Rush Medical College, Chicago.

1. *Bloodless Surgery of the Extremities.* By J. E. KELLY, L.R.C.S.D. (The Medical Press and Circular, Dec. 17, 1873.)

2. *On Laryngotomy.* By J. MORGAN, F.R.C.S. Read before the Surgical Society of Ireland, Jan. 16. (The Medical Press and Circular, Feb. 4, 1874.)

3. *Sarcoma Testis;* in which the testicle was retained for many years in the inguinal canal, but eventually descended into the scrotum, and in which, on removal, a portion of the omentum was found adherent to the diseased gland. By HENRY J. TYRRELL, F.R.C.S.D. (The Medical Press and Circular, Feb. 4, 1874.)

4. *A Case of Primary Excision of the Ankle Joint; with Observations.* By HENRY LEE, F.R.S. Transactions of Royal Medical and Chirurgical Society. (Medical Press and Circular, Feb. 11, 1874.)

5 *The Operation for Recto-Vaginal Fistula.* By H. T. WHITTELL, M.D. (London Lancet, Feb., 1874 )

6. *Tumor of Thigh, composed of Muscular Tissue.* Under the care of Mr. JNO. WOOD, King's College Hospital. (London Lancet, Feb., 1874.)

7. *Hereditary Exostoses.* Under the care of Dr. POORE, Charing-Cross Hospital. (London Lancet, Feb., 1874.)

8. *A New Plan in the Treatment of Erectile Tumors by Vaccination.* By Dr. P. BATHEDAT, of Bayonne. (London Lancet, Feb., 1874.)

9. *The Efficiency of Enemata.* (Philadelphia Medical Times, Feb. 7, 1874. Archiv. fur Klinische Chirurgie. Boston Medical and Surgical Journal.)

10. *Extirpation of the Larynx with the Epiglottis.* By Professor BILLROTH. (The Clinic, Feb. 28, 1874. British Medical Journal, Jan. 31, 1874.)

1. Dr. Kelly states that the operations upon which Esmarch's method confers the greatest advantages are those in which much blood is ordinarily lost, or those performed under circumstances which prevent the operator from providing efficient aid. Amongst the former, resections and explorations, either for foreign bodies or where preliminary investigations are judicious, may be specially noticed. These preliminary measures, so conservative in their aim and results, have often been frustrated by the danger or dread of consequent hæmorrhage. Against the obvious advantages of the expedient, we must, in justice, weigh some well-founded objections. In cases where collections of septic matter exist, Esmarch warns us against the danger of favoring by pressure its entrance into the circulation. The absence of bleeding points during the operation may cause us to neglect to secure some vessels which will subsequently be troublesome. As observed in amputations performed in Guy's and other hospitals, the edges of the incisions may slough.

(In a case of re-amputation at St. Luke's Hospital, recently, in which operation Esmarch's method was employed, the edges of the incision sloughed. It is not, however, our opinion that the employment of the above-mentioned method was productive of sloughing in this case. This process (sloughing) was confined to the old cicatrix, which closely resembled cartilage—just such tissue as will almost invariably slough away after re-amputation, which was demanded in consequence of sloughing of flaps after the primary amputation. O.)

I noticed this in my first case only, says Dr. Kelly, and my observations on it and the succeeding cases induce me to regard the sloughing as proportionate to and dependent on the *duration* of the pressure by the elastic bandage, and not to the *bloodless condition* of the tissues. I regard sloughing as unimportant in those cases which are really benefited by the method. In amputations and other operations which can be completed in a few minutes, I cannot recognize the necessity of departing from the old and efficient method of controlling hæmorrhage by direct arterial compression. Where the surgeon must necessarily amputate without sufficient skilled assistance, he is free to select between the immediate danger of hæmorrhage and the remote disadvantage of sloughing of the flaps. The quantity of blood lost during operation is frequently a most desirable antiphlogistic. By Esmarch's method, for good or for evil, this is prevented, and severe inflammation may follow the operation; however, in such cases the surgeon can resort to phlebotomy, or other antiphlogistic treatment. Again, in disease or predisposition to congestion in any of the important viscera, as the lungs or brain, we may well hesitate before we throw the entire amount of blood contained in a limb, either temporarily or permanently, into the general circulation. We must also remember the possibility that this quantity of blood may embarrass the pulmonary circulation, and aid in developing the fatal effects of the anæsthetic.

2. The paper treats of this operation (laryngotomy) as performed in cases of chronic syphilitic laryngeal disease, where the affection may either directly endanger life by its interference with respiration, or by wearing down the patient by its exhausting irritation.

3. The author states that in this case, which he is about to bring under the notice of the Society (Surgical Society of Ireland), a complication was found which, as far as he had been able to discover, had not before been placed on record. The subject of the communication was a farmer, aged 45 years, the father of five children. He had a large scrotal tumor on the left side, about the size of a small melon, heavy, smooth and elastic; the skin moved freely on it, it had no impulse on coughing, and the cord, though thickened, could be distinctly felt. There was no enlargement of the lumbar glands. It was not translucent. Dr. Tyrrell tapped the tumor with the aspirateur, and drew away half an ounce of arterial blood. A solid enlargement of the testicle was diagnosed, but the true nature of the enlargement was not made out. The patient stated that for many years he had no testicle on the left side in the scrotum, but a soft lump in the groin; that, when he was about twenty years of age, it descended into the scrotum and became painful; but that he was able to press it back again into the groin, where it would remain for a few days and descend again. During the last six years the tumor has grown larger and harder, and during this time he has been unable to press it back into its original position. The case lacks the history of injury or venereal taint.

On the 13th of last September he returned to Dublin, having been in the country, and was admitted into the Mater Misericordiæ Hospital. The testicle had increased much in size, and had become very inconvenient from its weight and size. At a consultation, Dr. Tyrrell's colleagues agreed with him that it was a sarcomatous tumor, and should be removed. Two other surgeons of great eminence saw the case, and coincided in opinion with Dr. Tyrrell, but none suspected the complication that was discovered at the operation.

Upon cutting down on the tumor, Dr. Tyrrell found a portion of the omentum in the tunica vaginalis, and intimately attached to the testicle. It was agreed to ligature this portion of the omentum with a carbolized gut ligature, and return the stump and ligature into the abdomen. The cord was tied en masse with a strong hempen thread, and the diseased gland and the portion of omentum attached was removed. No bad symptom appeared, and the wound quite healed twenty-five days after the operation. The tumor proved to be an example of spindle-celled sarcoma.

4. The author describes what he believes to be the only case in which complete primary section of the ankle joint has been performed, and advocates the plan, both in primary and secondary excisions of the joint, of dislocating the tibia and fibula outwards, so as to allow of the articulating surface of the tibia being removed with comparatively little disturbance to the surrounding parts. The articulating surface of the astragalus is also more easily removed in this way than by dislocating the bones of the leg inwards, as has commonly been attempted in secondary excisions. The plan advocated is to remove the internal malleolus first, and then the tibia and fibula may be dislocated outward through the external wound with great facility, and without interfering with any important structures. Such a mode of operating has not, he believes, been hitherto described.

5. Dr. Whittell (Hon. Medical Officer, Adelaide Hospital, South Australia), operated in the usual way, and, having brought the edges together with wire sutures, ordered opium to be given daily, with a view to prevent any action of the bowels. The sutures were removed after the lapse of ten days, when it was found that the operation had failed. Between two and three months subsequently, a second operation was performed, and this also failed. After these disappointments, the author began to question whether the usual plan of locking up the bowels with opium for several days after the operation, was not the real cause of failure. On each occasion the case progressed favorably during the first few days, and it seemed to be probable that the accumulation of fœcal matter, and its subsequent passage along the rectum in a hardened condition, tore open the tender structure by which the pared edges of the bowel were united, and thus spoiled the operation. The usual operation was performed for the third time. On this occasion, however, instead of locking up the bowels, castor oil sufficient to keep the bowels moderately relaxed, was ordered. Two or three fluid motions were passed daily without pain or inconvenience. When the sutures were removed, perfect union had taken place.

6. A butcher, aged 27 years, after hard dancing, suddenly felt something give way at the inner and upper part of the left thigh, and found a small lump had formed there. The accident was accompanied with nausea and subsequent occasional vomiting. When lying down the tumor disappeared, but returned when he assumed the erect position. At one of the London hospitals, two or three days after the patient discovered the tumor, an obturator hernia seems to have been diagnosed. Upon admission (to King's College Hospital), there was a tumor about the size of a hen's egg, at the inner and upper part of the front of the left thigh, one inch and a half below Poupart's ligament, to the inner side of the saphenous opening, which was quite free. The tumor was apparently situated immediately upon the adductor longus muscle. It was elastic and non-fluctuating; there was no impulse on coughing. The skin was freely movable over it, but the tumor could be isolated, or moved independently of the deeper parts. The patient had a constant pain all down the inner side of the same thigh, extending over the left buttock and loin. The tumor was largest when the knee was flexed, and the thigh maintained by the patient in an everted position; but when the thigh was supported, and the patient told to allow the limb to drop, and thus to relax the muscles, the tumor entirely disappeared, but when told to adduct the thigh, the tumor stood out again as before.

Mr. Wood finally made an incision over the tumor, and carefully divided the fascia, and immediately came upon a mass of muscular tissue, of which the tumor was composed, and it was evident that a hernial protrusion of a portion of the muscular belly of the adductor longus had taken place, through weakening of its aponeurosis, and thus given rise to the tumor.

7. A clerk aged 27, was studded with bony tumors, ranging in size from a small marble to an orange. The patient's little daughter, aged four and a half years, was a complete miniature of her father, and presented exostoses in most of the situations where they were observable in him. Her first exostosis was noticed when she was nine months old. The patient states that his maternal grandfather presented the same phenomena. One of his sisters, aged thirty, has a tumor on the lower end of the femur.

8. Dr. Bathedat, in a letter addressed to the editor of L'Union Medicale, points out what he supposes to be the cause of frequent failures attending the treatment of erectile tumors by vaccinations. The drop of blood that follows the prick of an ordinary lancet, carries away the vaccine lymph, and very few or no pustules are obtained. To remedy this he uses a needle instead of a lancet. The punctures can be multiplied *ad infinitum*, so that a veritable vaccine crust is formed over all the diseased surface, whatever may be its extent. Tumors, he adds, the dimensions of which would not even have allowed a trial of cure with the ordinary lancet, may be treated by this method with a fair chance of success.

9. Gustav Simon has succeeded in demonstrating that a stream of water forced into the rectum by means of a syringe, may be made to penetrate the entire length of the large intestine, and possibly extend also into the small intestine. His experiments were performed upon two separate patients, each of whom happened to have a fistulous opening in the ascending colon, near its junction with the cæcum.

10. Professor Billroth has performed the operation of removing the larynx and epiglottis. The patient was a strong man about forty years of age, the subject of cancerous growths in the larynx, which had repeatedly, after putting him in danger of death from suffocation, been removed by Dr. Storck, with the aid of the laryngoscope. Prof. Billroth's operation was performed on the last day of 1873. In the beginning of November, the new growths extended so far into the interior of the larynx, that their removal from above was no longer possible. As a part of the right vocal cord was present, Drs. Storck and Billroth hoped to preserve this, however imperfect; they therefore opened the larynx from the front, and, after removing the growths, applied solution of perchloride of iron to the inner surface. The result of this operation appeared at first to be very promising, but in the middle of December new growths were detected, and at the end of the month symptoms of asphyxia. Drs. Billroth and Storck decided that the whole larynx was so full of malignant growths that it would be useless to repeat the operation of dividing it and cleaning them out, and there was no longer a possibility of preserving any part of the vocal cord. Extirpation of the part would produce no additional physiological defect, and might lead to a radical cure if the disease was confined to the part, and had not reached the glands. Professor Billroth therefore removed the entire larynx. The patient bore the operation very well; he breathed freely through the trachea, in which a tube was inserted; the fever was slight, and of short duration; and on January 9th the wound was healing favorably. On the 24th the man was reported to be able to eat and drink, and to sit up for several hours daily.

